# A case report of thyroid-associated Orbitopathy with elevated TPO antibodies

**DOI:** 10.1186/s12902-020-00658-6

**Published:** 2020-11-27

**Authors:** Radwan El Othman, Christelle Ephrem, Elsie Touma, Souheil Hallit, Rola El Othman

**Affiliations:** 1 Faculty of Medicine and Medical Sciences, Holy Spirit University of Kaslik (USEK), Jounieh, Lebanon; 2 Department of Internal Medicine, University Hospital Center- Notre Dame des Secours, Byblos, Lebanon; 3 INSPECT-LB: Institut National de Sante Publique, Epidemiologie Clinique et Toxicologie-Liban, Beirut, Lebanon; 4 Bahman Hospital, Beirut, Lebanon

**Keywords:** Hashimoto’s thyroiditis, Hashimoto’s disease, Orbitopathy, Ophtalmopathy, Case report

## Abstract

**Background:**

Thyroid associated orbitopathy (TAO) is defined as an immune mediated inflammatory process affecting the extraocular muscles, connective and adipose tissue of uncertain etiopathogenesis. TAO are classically described in Grave’s disease (GD) however it may occur in euthyroid and hypothyroid patients. Those patients usually test positive for Thyroid Stimulating Hormone receptor antibodies (TRAb). For instance, only few cases of severe Hashimoto’s thyroiditis (HT) associated orbitopathy with negative TRAb are reported to date.

**Case presentation:**

Herewith we report a rare case of a middle-aged female who presented with bilateral progressive upper and lower palpebral edema and a unilateral marked proptosis associated with asthenia, headache and decrease in visual acuity. Biological investigation was notable for high levels of anti-thyroid peroxidase antibodies (Anti-TPO) in an otherwise euthyroid patient with negative TRAb. Orbital Magnetic resonance imaging revealed edema of the extraocular muscles and inflammation of periorbital soft tissue. The patient received a treatment with intravenous methylprednisolone followed by oral treatment with prednisone. This regimen was both effective and safe with minimal metabolic side effects in our patient.

**Conclusion:**

Minor ocular manifestations of HT are common; however, severe sight threatening ophtalmopathy in the absence of TRAb is rare. Multiple differential diagnosis should be considered and investigated before diagnosing this rare entity. Management of similar cases is currently based on reports and no clear guidelines have been elaborated, corticosteroids is the mainstream of treatment with a potential benefit of selenium supplementation in mild to moderate cases.

## Background

Thyroid associated orbitopathy (TAO) is commonly defined as an immune mediated inflammatory process affecting the extraocular muscles, connective and adipose tissue of uncertain etiopathogenesis [1]. Common findings are periocular tissue edema, decreased visual acuity, ocular pain and exophthalmos; negatively impacting patient’s quality of life. In severe cases, inflammatory mediated optic nerve compression may cause loss of vision. Both eyes are usually affected with only 10–14% of patients having unilateral ocular involvement. TAO are classically described in Grave’s disease however it may occur in euthyroid and hypothyroid patients; accounting for 4.3% of all TAO [2]. Those patients usually test positive for Thyroid Stimulating Hormone receptor antibodies (TRAb) and are thus diagnosed to have euthyroid or hypothyroid GD. Several studies have revealed the correlation between TAO and serum TRAb levels; however, a different mechanism is likely to be behind this rare manifestation of Hashimoto’s Thyroiditis or euthyroid patients with negative TRAb [1]. Although upper eyelid retraction has been described as a common feature of Hashimoto’s thyroiditis; severe orbitopathy associated with Hashimoto’s thyroiditis has been rarely reported in the literature with limited clinical experience regarding its management and outcome. Herewith we report a case of middle-aged woman who initially presented with euthyroid associated orbitopathy and elevated TPO antibodies and subsequently developed subclinical hypothyroidism.

## Case presentation

**Fig. 1:**
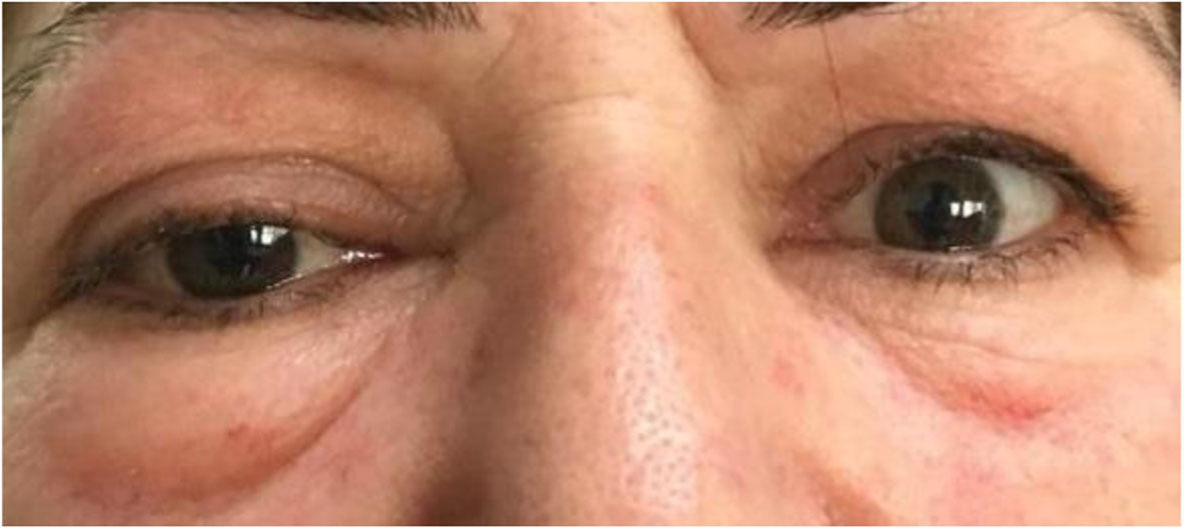
Patient at presentation with palpebral edema and proptosis

A 52-year old unemployed Caucasian female was referred by her general physician to internal medicine and clinical immunology clinic for a ten-month history of progressive tender to touch bilateral upper and lower palpebral edema, affecting mainly the right eye (Fig. 1). Edema was associated with an upper eye lid retraction and a marked proptosis mainly to the left. In her medical history, the patient is known to have dyslipidemia for which she is receiving 5 mg of Rosuvastatin daily and 1.5 mg of Bromazepam daily for an old unspecific anxiety disorder for which she is not receiving constant psychiatric follow-up. The patient is known to have a single kidney to the right following a non-functional left kidney nephrectomy in childhood. No past personal history of exposure to radioactive iodine is noted nor familial history of thyroid diseases. The patient reported an associated asthenia, anorexia, a recent bilateral decrease in visual acuity mainly affecting the right eye, blurring and double vision along with vertigo and a chronic tension headache. The patient is a heavy smoker (40 cigarettes daily for more than 30 years). Patient was overweight with a Body mass index (BMI) of 26.9 kg/m^2^. Physical examination was particular for an ocular proptosis mainly in the left eye, low grade pain on orbital palpation and a multinodular hypertrophic thyroid gland. Ophthalmological examination revealed bilateral keratoconus especially in the right eye with an ipsilateral decrease in visual acuity and a clinical activity score (CAS) of 3 at presentation. Physical examination including neurological examination was otherwise normal.

## Investigations

Patient was admitted to the hospital for further investigation and management. Initial blood investigations showed normal complete blood count, C reactive protein level, erythrocyte sedimentation rate, procalcitonin, electrolytes, BUN, serum creatinine, liver biochemistry and serum protein electrophoresis. Fasting lipid profile was as follows: Triglyceride 144 mg/dL, total cholesterol 163 mg/dL, Low-density lipoprotein (LDL) 103 mg/dL and High-density lipoprotein (HDL) 44 mg/dL.

**Fig. 2:**
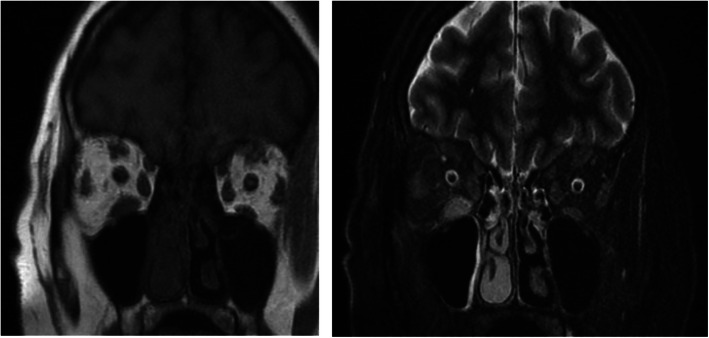
Orbital MRI at presentation showing tumefaction and edema of the extraocular muscles of the orbit (right inferior and right lateral muscle) with inflammation of the periorbital soft tissue

The initial biological investigation was followed by a cerebral and orbital 1.5 Tesla Magnetic Resonance Imaging (MRI) under sedation for claustrophobia, which revealed a tumefaction and edema of the extraocular muscles including the right inferior and right lateral muscle and to a lesser extent the left inferior orbital muscle with slight infiltration of the orbital fat and inflammation of the periorbital soft tissue especially to the right (Fig. 2). Optical nerves were of normal thickness and signal. Imagery findings were suggestive of an orbital inflammatory pseudo-tumor.

Based on the initial work-up four major differential diagnosis were investigated: sarcoidosis, tuberculosis, thyroid associated orbitopathy and a lymphoproliferative process.

In order to make a definitive diagnosis a panel of additional laboratory examinations was performed and is summarized in Table 1. Furthermore, a high resolution chest computerized tomography (CT) with contrast was performed and revealed no argument in favor of sarcoidosis.

**Table 1:** Results summary of biological markers studied upon presentation

	Value	Reference Range
Angiotensin converting enzyme (ACE)	35.8 U/L	8–65 U/L
Tuberculin skin test (TST)	< 5 mm	0–5 mm: Negative
Serum proteins electrophoresis	Normal pattern	
Thyroid stimulating hormone (TSH)	0.615 μIU/mL	0.27–4.2 μIU/mL
Free thyroxine (F T4)	14.05 pmol/L	12–22 pmol/L
Free triiodothyronine (F T3)	2.94 pmol/L	3.1–6.8 pmol/L
**Anti-thyroid peroxidase (Anti-TPO)**	**855.28 IU/mL**	**0–5.6 IU/mL**
TSH receptor antibodies (TRAb) by enzyme immunoassay	< 0.6 IU/L	Negative: < 1 IU/L
Anti-nuclear antibodies by immunofluorescence (ANA by IF)	< 1/100	< 1/100: Negative
Perinuclear anti-neutrophil cytoplasmic antibodies (P-ANCA)	< 20 RU/ML	< 20 RU/ML: Negative
Cytoplasmic anti-neutrophil cytoplasmic antibodies (C-ANCA)	< 20 RU/ML	< 20 RU/ML: Negative
IgG4 - Nephelometry	151 mg/dL	3–201 mg/dL

The diagnosis of thyroid associated orbitopathy was made; thyroid gland echography showed a polylobulated thyroid gland with heterogeneous echostructure and multiple solid nodules along with inflammatory regional lymph nodes.

## Treatment, outcome and follow-up

An urgent high dose intra-venous (IV) steroid therapy was administered at day 2 of hospitalization for 4 days at a dose of 0.5 mg/Kg of methylprednisolone twice daily which resulted in significant reduction in palpebral edema and limited the progression of the decrease in visual acuity. IV steroid therapy was followed by 2 months treatment with oral prednisone starting with 60 mg daily with progressive tapering of half dose every 2 weeks. Patient was instructed to quit smoking due to its deleterious effect on her orbitopathy; however, the patient only managed to reduce her tobacco consumption to 20 cigarettes daily over a period of 2 months.

**Fig. 3:**
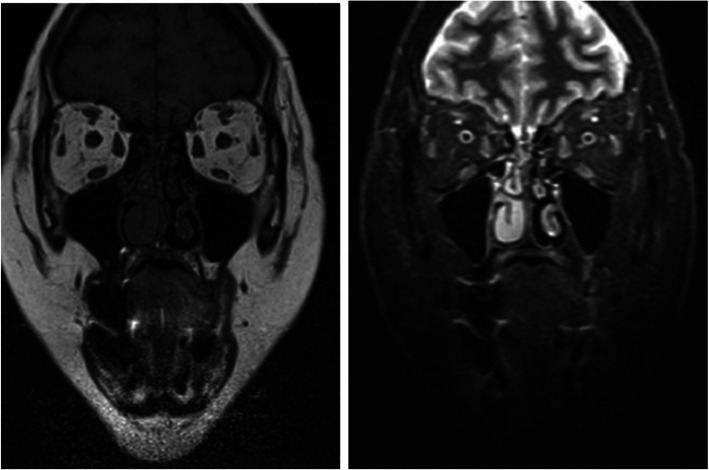
Follow-up orbital MRI performed upon completion of oral therapy; showing resolution of the edematous anomaly of the extraocular muscles and regression of proptosis to the right

**Fig. 4:**
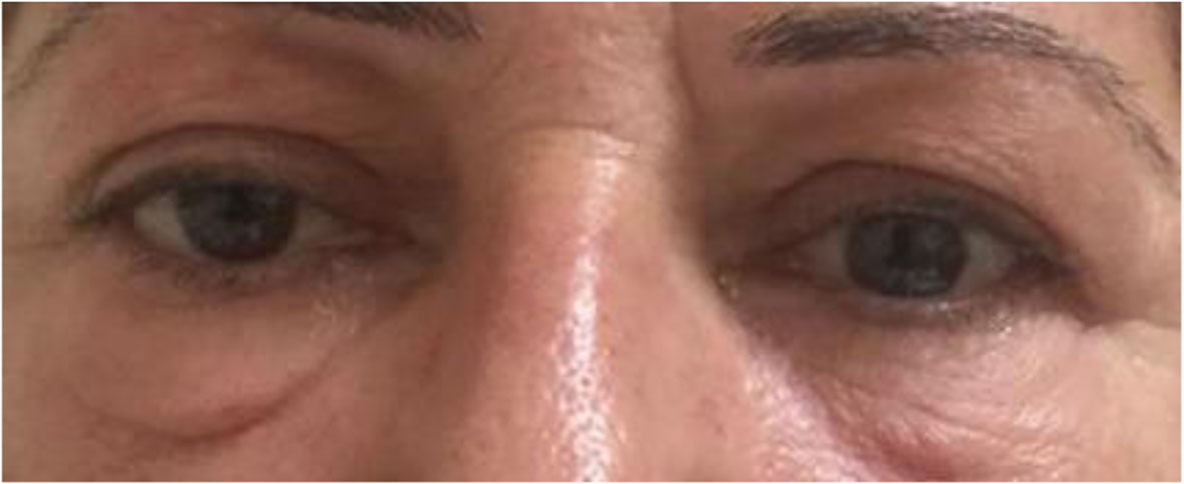
Patient at 6 months follow-up visit with a marked resolution of proptosis and palpebral edema

Patient was regularly followed every 2 weeks to ensure compliance, assess evolution and determine potential side effects. Upon the completion of 2 months treatment with oral prednisone a marked regression of the palpebral edema was noted with no further deterioration in her visual acuity. An orbital MRI was performed upon completion of oral therapy and showed a resolution of the edematous anomaly of the right lower muscle, absence of extraocular muscles signal abnormality with symmetrical globes and regression of the grade II proptosis to the right (Fig. 3). In addition, biological markers evaluation was performed upon the completion of oral therapy and showed minor metabolic side effects of the treatment namely a mild elevation in total cholesterol and LDL levels (Table 2). Furthermore, thyroid function evaluation upon completion of treatment was notable for a slight increase in TSH with normal FT3 and FT4 (Table 2). Even though, steroids are usually associated with a decrease in TSH level through its effect on TRH [3]. However, in our case TSH level slightly increased after completion of steroids treatment with normal FT4 falling in subclinical hypothyroid range; hence, suggesting the diagnosis Hashimoto’s Thyroiditis. Patient was scheduled to have a follow-up visit every 3 months for the next 1 year in order to monitor for development of overt hypothyroidism requiring further treatment. No weight changes were recorded. At 6 months follow-up patient was symptom free with no ocular proptosis, no further deterioration in her visual acuity beyond the baseline and a minor residual right palpebral edema (Fig. 4) with a clinical activity score of 1. The patient equally reported an improvement in her asthenia and a reduction in frequency of her tension headaches.

**Table 2:** Results summary of biological markers studied upon completion of treatment

	Value	Reference Range
Hemoglobin A1C	5.7%	< 6%
Total Cholesterol	212 mg/100 mL	120–220 mg/100 mL
High Density Lipoprotein (HDL)	45 mg/100 mL	45–64 mg/100 mL
Low Density Lipoprotein (LDL)	139 mg/100 mL	80–150 mg/100 mL
Triglycerides	152 mg/100 mL	50–200 mg/100 mL
Thyroid stimulating hormone (TSH)	4.83 μIU/mL	0.45–4.5 μIU/mL
Free thyroxine (F T4)	1.25 ng/100 mL	0.7–1.85 ng/100 mL
Total triiodothyronine (T T3)	1.2 ng/mL	0.8–2.02 ng/mL

## Discussion and conclusions

Thyroid associated orbitopathy is far more common in Grave’s disease accounting for 90% of cases [4]. Its pathophysiology has been extensively studied and it’s related to TRAb that is expressed on both thyroid follicular cells and orbital fibroblasts [5, 6]. In fact, the autoimmunity affecting the receptors on orbital tissues causes local inflammation, hyaluronic acid deposition and expansion of local adipose tissue eventually leading to connective tissues remodeling and fibrosis in advanced stages [7–9]. However, only few reports address the cases of severe orbitopathy associated with euthyroid or hypothyroid state. The largest study regarding the prevalence of orbitopathy among HT patients was conducted by Kahaly and al. on 700 patients and revealed that clinically significant TAO was present in only 6% of cases [10] additionally the study revealed that patient with Hashimoto’s disease associated orbitopathy were heavy smokers and less likely to suffer from other autoimmune disease in comparison with patient with HT without TAO. Similarly our patient is a heavy smoker and was not diagnosed to have another autoimmune disease. The TRAb theory may explain the presence of TAO in HT patients with positive TSAb (Thyroid Stimulating Antibody) a subtype of TRAb. In fact, Kahaly and al. found a positive correlation between the levels of TSAb and the severity of TAO. Additionally, their work has showed that only 5.5% of patient with HT and negative TSAb had clinically overt orbitopathy versus 68% with positive TSAb [10]. Hence, further emphasizing the implication of TSAb/TRAb is the pathogenesis of TAO. However, the pathogenesis of TAO in patients with negative TRAb remains unknown. One hypothesis is that autoimmunity is directed toward calsequestrin in extraocular skeletal muscles and against collagen XIII in orbital fibroblasts [9]. Furthermore, several studies have shown that low vitamin D was correlated with abnormal thyroid function tests and the presence of antithyroid antibodies [11–13]. In one retrospective study, Lahooti et al. concluded that vitamin D deficiency was a risk factor for TAO in GD, but not in HT. Thus suggesting a potential different pathogenesis [14].

Unilateral exophthalmos has a wide set of differential diagnosis including orbital primary and secondary neoplasms (e.g. lymphoma, rhabdomyomas, metastasis), specific orbital inflammation (e.g. sarcoidosis), infections, vascular etiologies, myositis, orbital pseudotumor, IgG4-related diseases, TAO … [15–17] A gold standard for diagnosis is Magnetic resonance imaging a noninvasive imaging allowing evaluation of soft tissue with great accuracy [18].

Ocular manifestations of thyroid diseases are frequent with upper eyelid retraction being the most common ocular sign in HT and retrobulbar pain the most prevalent ocular symptom [19, 20]. For instance, severe TAO among HT patients is a rare finding. Most reported cases describes patients with bilateral TAO rather than unilateral ocular involvement and mostly with positive TRAb and an excellent response to glucocorticoids [21–23]. Therapy with intravenous corticosteroids represent the cornerstone in the management of severe TAO with a good response rate of 70–80% versus 50–60% for oral therapy with fewer side effects for short-term IV regimen [24–26]. According to the European Group on Graves’ Orbitopathy the most common schedule for IV steroid treatment is a cumulative dose of 4.5 g of methylprednisolone divided over 12 weeks (0.5 g/week for the first 6 weeks followed by 0.25 g/week for the second 6 weeks) without exceeding a cumulative dose of 8 g per 12 weeks [27]. In addition, studies have shown that adding selenium to the treatment regimen of mild to moderate orbitopathy was effective in reducing local inflammation, decrease Anti-TPO production and improve thyroid morphology [28]. Due to the limited number of cases of HT associated orbitopathy, no conventional guidelines exist regarding its management; most reported cases were treated with 1 to 5 g of methylprednisolone during the 1st week followed by a tapered glucocorticoid dose over several weeks [21–23]. Our patient has received a reduced dose of IV methylprednisolone equivalent to a cumulative of 0.3 g infused over a period of 4 days followed by 2 months of oral prednisone treatment starting with 60 mg daily and tapered by half every 2 weeks. This regimen was both effective and safe with minimal metabolic side effects in our patient.

In conclusion, minor ocular manifestations of HT are common; however, severe orbitopathy in the absence of TRAb is rare and should be diagnosed early in the evolution of the disease in order to prevent sight threatening complications. In the present we are reporting a case of moderate to severe orbitopathy associated with HT a rare entity to date especially with negative TRAb. Multiple differential diagnosis should be considered and investigated before concluding the presence of this rare entity. MRI of the orbit remains the cornerstone in the diagnosis. Management of similar cases is currently based on reports and no clear guidelines have been elaborated, corticosteroids following various regimen is the mainstream of treatment with a potential benefit of selenium supplementation in mild to moderate orbitopathy. Further studies are warranted to determine the exact pathogenesis of this rare manifestation of TRAb negative HT especially that TPO antibodies are equally present in 80% of GD patients [29] thus suggesting an involvement of these antibodies in the pathophysiology of orbitopathy in both GD and HT.

## Data Availability

All data pertaining to this patient are included in this report.

## Abbreviations

TAO: Thyroid associated orbitopathy
GD: Grave’s disease
TRAb: Thyroid Stimulating Hormone receptor antibodies
HT: Hashimoto’s thyroiditis
Anti-TPO: Anti-thyroid peroxidase antibodies
BUN: Blood urea nitrogen
LDL: Low-density lipoprotein
HDL: High-density lipoprotein
MRI: Magnetic Resonance Imaging
CT: Computerized tomography
IV: Intra-venous
ACE: Angiotensin converting enzyme
TST: Tuberculin skin test
TSH: Thyroid stimulating hormone
F T4: Free thyroxine
F T3: Free triiodothyronine
ANA: Anti-nuclear antibodies
IF: Immunofluorescence
P-ANCA: Perinuclear anti-neutrophil cytoplasmic antibodies
C-ANCA: Cytoplasmic anti-neutrophil cytoplasmic antibodies
TSAb: Thyroid Stimulating Antibody
